# P48 The limited yield of cultures in the critically ill child

**DOI:** 10.1093/jacamr/dlac004.047

**Published:** 2022-02-16

**Authors:** John Clark, Deborah White, Esther Daubney, Martin Curran, Rachel Bousfield, Theodore Gouliouris, Elizabeth Powell, Adam Palmer, Shruti Agrawal, David Inwald, Iain Kean, M. Estée Török, Stephen Baker, Nazima Pathan

**Affiliations:** Department of Paediatrics, University of Cambridge, Cambridge, Cambridgeshire, CB2 0QQ, UK; Cambridge University Hospitals NHS Foundation Trust, Cambridge, Cambridgeshire, CB2 0QQ, UK; Cambridge University Hospitals NHS Foundation Trust, Cambridge, Cambridgeshire, CB2 0QQ, UK; Cambridge University Hospitals NHS Foundation Trust, Cambridge, Cambridgeshire, CB2 0QQ, UK; Clinical Microbiology and Public Health Laboratory, Public Health England, Cambridge, Cambridgeshire, CB2 0QQ, UK; Cambridge University Hospitals NHS Foundation Trust, Cambridge, Cambridgeshire, CB2 0QQ, UK; Cambridge University Hospitals NHS Foundation Trust, Cambridge, Cambridgeshire, CB2 0QQ, UK; Cambridge University Hospitals NHS Foundation Trust, Cambridge, Cambridgeshire, CB2 0QQ, UK; Cambridge University Hospitals NHS Foundation Trust, Cambridge, Cambridgeshire, CB2 0QQ, UK; Cambridge University Hospitals NHS Foundation Trust, Cambridge, Cambridgeshire, CB2 0QQ, UK; Cambridge University Hospitals NHS Foundation Trust, Cambridge, Cambridgeshire, CB2 0QQ, UK; Department of Paediatrics, University of Cambridge, Cambridge, Cambridgeshire, CB2 0QQ, UK; Cambridge University Hospitals NHS Foundation Trust, Cambridge, Cambridgeshire, CB2 0QQ, UK; Department of Medicine, University of Cambridge, Cambridge, Cambridgeshire, CB2 0QQ, UK; Cambridge Institute of Therapeutic Immunology and Infectious Disease, University of Cambridge, Cambridge, Cambridgeshire, CB2 0QQ, UK; Cambridge Institute of Therapeutic Immunology and Infectious Disease, University of Cambridge, Cambridge, Cambridgeshire, CB2 0QQ, UK; Department of Paediatrics, University of Cambridge, Cambridge, Cambridgeshire, CB2 0QQ, UK; Cambridge University Hospitals NHS Foundation Trust, Cambridge, Cambridgeshire, CB2 0QQ, UK

## Abstract

**Background:**

Broad-spectrum antimicrobial therapy is a key driver of antimicrobial resistance. Here, we aimed to review indications for antimicrobial therapy, determine the proportion of suspected bacterial infections that are confirmed by culture, and assess the time taken for microbiology test results to become available in the paediatric ICU (PICU).

**Methods:**

A single-centre prospective observational cohort study of 100 consecutive general PICU admissions from 30 October 2019 to 19 February 2020. Data were collected from the hospital medical record and entered into a study database prior to statistical analysis using standard methods.

**Results:**

Of all episodes of suspected infection, 22% of lower respiratory tract infection, 43% of bloodstream and 0% of central nervous system infection were associated with growth on microbiology culture. Ninety percent of children received antimicrobial therapy. Hospital-acquired infection occurred less commonly than primary infection, but an organism was grown in a greater proportion (64%) of cultures. Final laboratory reports for negative cultures were issued at a median of 120.3 h for blood cultures and 55.5 h for endotracheal tube aspirate cultures.

**Conclusions:**

Despite most critically children receiving antimicrobial therapy, infection was often not microbiologically confirmed. Novel molecular diagnostics may improve rationalization of treatment in this population.

